# Impact on health and well-being of working at home during the SARS-CoV-2 pandemic

**DOI:** 10.47626/1679-4435-2022-791

**Published:** 2022-03-30

**Authors:** Alberto José Niituma Ogata, Ana Maria Malik, Viviane Lourenço, Valena Savia, Ana Claudia Pinto, Yohana Rodrigues

**Affiliations:** 1 Centro de Estudos em Planejamento e Gestão de Saúde - FGVsaúde, Escola de Administração de São Paulo, Fundação Getulio Vargas, São Paulo, SP, Brazil.; 2 MBA Gestão de Programas de Promoção da Saúde nas Organizações, Centro Universitário São Camilo, São Paulo, SP, Brazil.; 3 Hospital das Clínicas da Faculdade de Medicina da Universidade de São Paulo, São Paulo, SP, Brazil.

**Keywords:** working environment, coronavirus infections, mental health, occupational health

## Abstract

**Introduction:**

After the onset of the severe acute respiratory syndrome coronavirus 2 (SARS-CoV-2) pandemic, many workers were forced to start working from home, creating a new dynamic that could potentially affect their health in several ways.

**Objectives:**

To study the impact of working at home during the SARS-CoV-2 pandemic on a sample of Brazilian workers.

**Methods:**

This study used a cross-sectional methodology with an online survey conducted by a Brazilian human resources website from June 1 to August 15, 2020, with a sample of employees working at home during the SARS-CoV-2 pandemic.

**Results:**

The sample of 653 valid responses revealed that 87.7% of the survey respondents reported that the change to home working started because of the situation caused by the pandemic. However, 550 (84.2%) people from this group stated that their employer did not conduct any health and safety evaluation of their workstation in the domestic environment. Regarding physical symptoms, there were high prevalence rates of symptoms related to musculoskeletal conditions, sleeping problems, feelings of fatigue, headaches, and migraines. The study also used the World Health Organization-5 Well-Being Index instrument and there were statistically significant associations between low scores and physical symptoms of musculoskeletal conditions, feelings of fatigue, headache or migraine, heartburn and indigestion, and leg pain.

**Conclusions:**

The findings of this research confirm the importance of developing strategies and programs to preserve the health and well-being of workers who start working at home, with participation of and supervision by companies’ occupational physicians. Future investigations should continue to capture data about health, well-being, and productivity and share best practices to plan support for the occupational health of those working from home.

## Introduction

After the onset of the pandemic caused by the severe acute respiratory syndrome coronavirus 2 (SARS CoV 2) virus, at the beginning of 2020, quarantine was declared in most parts of the world, including Brazil. In this context, many workers were forced to start working from home, to curtail physical and social contact among people and reduce new infections. Information technology and the Internet were some of the factors that enabled implementation of this measure.

The need to work from home made a new dynamic necessary, with online meetings and phone or video calls and innovative ways of conducting tasks and organizing work. This has led to substitution of traditional events, oftentimes without the training and preparation for the change that would be desirable.^1^ Employees miss the sociability and benefits of collaboration offered by working in shared workspaces.^2^

The unusual circumstances of lockdown meant people were often working at home for the first time, adapting to the new practice without warning. They might be sharing their home-based workspace with others, and therefore have little privacy, or may have to be peripatetic, negotiating their use of space around household members’ relative need for quiet.^2^

The domestic environment does not provide the adequate furniture for work, such as most companies provide. Besides, it also presents a range of factors that may interfere with working activities, thus potentially favoring the emergence or aggravation of pre-existing conditions such as musculoskeletal pain, stress, anxiety, and fatigue. Moreover, the change can affect lifestyle, with alterations to eating habits and physical activity patterns.

Considering the individual level, some authors have proposed suggestions for maintaining well-being, including: create routines, be organized, have an adequate home office environment, enhance one’s productivity, be responsible, avoid extreme multitasking, facilitate communication and networking, be balanced, use available computer programs and platforms, be creative with remote teaching, explore options for remote research, and learn from the challenges.^3^ However, several studies have shown effects on rates of musculoskeletal pain, sleep disorders, headaches, anxiety, depression, and gastrointestinal problems associated with changes in work processes during the SARS CoV 2 pandemic.^4-7^

A longitudinal study involving desk workers during shelter-at-home restrictions revealed an increase in non-workday sedentary behavior, worsening of sleep quality, increase in mood disturbance, and some decrease in perceived quality-of-life.^8^ A secondary analysis of a longitudinal cohort study conducted in the United Kingdom demonstrated that, during the lockdown, the population prevalence of clinically significant levels of mental distress rose from 18.9% (95% confidence interval [95%CI] 17.8-20.0) in 2018-19 to 27.3% (26.3-28.2) in April 2020.^9^ A cross-sectional study comparing changes occurred from before to during the SARS CoV 2 pandemic in Brazil revealed that there was a reduction in physical activity and vegetable consumption, as well as increases in the time spent using television and computers/tablets and in consumption of frozen foods, snacks, and chocolate.^10^

In this context, it is important to study these changes to understand their consequences, enabling preventive and corrective measures to be taken to reduce their impact on workers’ health.

## Methods

This is a cross-sectional tudy of a sample of Brazilian workers who started working from home during the SARS CoV 2 pandemic.

Initially, a questionnaire^11^ designed by the Institute of Employment Studies (IES), which formally authorized its use for this study, underwent a process of cross-cultural adaptation, including translation from English to Portuguese, reconciliation of translations, analysis by a group of specialists, back translation, and composition of the final version. The questionnaire incorporates the World Health Organization 5 - Well-Being Index (WHO-5, see Annex 1), an instrument already validated for use in Portuguese.^12-16^ This instrument is available in 30 languages and allows quick evaluation of individuals’ mental health and well-being conditions (mood, vitality, and general interest). Scores can vary from 0 to 25. Total scores below 13, besides showing low levels of well-being, highlight the need for monitoring, as they may signal some level of depression (with sensitivity of 93% and specificity of 83%).^12^

The questionnaire, in electronic format, was distributed through a human resources information portal (RH para você) and made available on social media between June 1 and August 15, 2020.

Quantitative data were collected via the survey consolidation method. The questionnaires were consolidated in MS-Excel sheets, taking care to maintain the identity of participants and their data confidential. Subsequently, the database was processed with R^®^ software. Qualitative answers were categorized for group analysis. Furthermore, the study population was presented and characterized according to an exploratory analysis using the chi-square test of independence to test for relations between the WHO-5 well-being score and the variables selected.

The study was approved by the Committee of Ethical Conformity Regarding Human Beings (CEPH) at the Fundação Getulio Vargas - 063/2020.

## Results

The survey was administered in online form between June 1 and August 15, 2020. A total of 653 valid answers were received in this period. Regarding the demographic profile, 436 (66.8%) of the respondents were female, with an average age of 40 years (median of 39 years and standard deviation [SD] of 10.3). Amongst these participants, 304 (46.6%) claimed to live with dependent children under the age of 18 years and 148 (22.7%) took care of another adult or elderly relative. In this context, 298 (45.6%) reported sharing their working space with another adult.

Most respondents (573, or 87.7% of the total) stated they had started working from home because of the SARS CoV 2 situation. However, 550 (84.2%) people from this group acknowledged that the employer had not conducted any health and safety evaluation of their workstation in the domestic environment.

Regarding physical symptoms, the high prevalence of symptoms related to musculoskeletal conditions should be highlighted. Most respondents mentioned that the following conditions now occur with a slightly higher or much higher frequency than usual: discomfort or back pain (366 respondents, 56% of the total), neck pain (362 respondents, 55.4% of the total) and shoulder pain (328 respondents, 50.3% of the total) (Table 1).

**Table 1:** Physical symptoms acknowledged during the confinement and home office period

Frequency	Lost sleep worrying about things		Neck pain*		Shoulder pain*		Back pain*	
	n	%	n	%	n	%	n	%
Not at all	137	21.0	126	19.3	167	25.6	129	19.8
No more than usual	158	24.2	165	25.3	158	24.2	158	24.2
Slightly more than usual	219	33.5	219	33.5	210	32.2	189	28.9
Much more than usual	139	21.3	143	21.9	118	18.1	177	27.1

* Discomfort, mild to intense pain.

Other symptoms were commonly felt, with a slightly or much higher frequency than usual, such as sleeping problems (358 respondents, 54.8% of the total), tired eyes (295 respondents, 45.2% of the total), feelings of fatigue (284 respondents, 43.5% of the total), and headaches and migraine (278 respondents, 42% of the total) (Tables 2 and 3).

**Table 2:** Physical symptoms acknowledged during the confinement and home office period

Frequency	Elbow pain*		Wrist and hand pain*		Hip pain*		Ankle pain*		Tired eyes	
	n	%	n	%	n	%	n	%	n	%
Not at all	372	57.0	282	43.2	281	43.0	357	54.7	201	30.8
No more than usual	139	21.3	163	25.0	126	19.3	151	23.1	157	24.0
Slightly more than usual	88	13.5	137	21.0	138	21.1	94	14.4	171	26.2
Much more than usual	54	8.3	71	10.9	108	16.5	51	7.8	124	19.0

* Discomfort, moderate to intense pain.

**Table 3:** Physical symptoms acknowledged during the confinement and home office period

Frequency	Headache or migraine		Chest pain		Leg cramps		Heartburn or indigestion		Fatigue	
	n	%	n	%	n	%	n	%	n	%
Not at all	195	29.9	456	69.8	440	67.4	285	43.6	219	33.5
No more than usual	180	27.6	98	15.0	124	19.0	154	23.6	150	23.0
Slightly more than usual	163	25.0	65	10.0	57	8.7	130	19.9	165	25.3
Much more than usual	115	17.6	34	5.2	32	4.9	84	12.9	119	18.2

The emotional issues most often reported with a slightly or much higher frequency than usual were worrying about family finances (234 respondents, 35.8% of the total), anxiety about a family member’s health (197 respondents, 30.1% of the total), and feelings of isolation and loneliness (75 respondents, 11.5% of the total) (Table 4).

**Table 4:** Emotional issues acknowledged during the confinement and home office period

Frequency	I have been worried about the family finances		I have been anxious about a family member’s health		I feel lonely and isolated	
	n	%	n	%	n	%
Never	33	5.1	79	12.1	309	47.3
Some of the time	194	29.7	204	31.2	154	23.6
Less than half of the time	84	12.9	75	11.5	62	9.5
More than half of the time	108	16.5	98	15.0	53	8.1
Most of the time	110	16.8	104	15.9	48	7.4
All the time	124	19.0	93	14.2	27	4.1

The instrument used, WHO-5, assesses subjective well-being based on mood, vitality, and general interest, considering a score of 13 (on a scale from 0 to 25 points) as an indicator of low level of well-being and suggesting there should be closer monitoring. The mean score in the study sample was 13.81 (SD of 5.88) and 298 respondents (45.63% of the total) had a WHO-5 score less than or equal to 13.

There were statistically significant associations between low perceived well-being level (observed by low scores on the WHO-5 questionnaire) and physical symptoms of musculoskeletal conditions (pain in the back, shoulders, wrists, hands, hips, ankles, and feet), feelings of fatigue, headache or migraine, heartburn and indigestion, and leg pain (Table 5). Furthermore, low perceived well-being level was more frequent in females (49% of the total, p = 0.031) and single individuals living alone (54% of the total, p = 0.007).

**Table 5:** Low perception of well-being (World Health Organization-5 Well-Being Index with score below 13) and physical complaints

Symptom*	n	%	p-value
Ankle and feet pain	43	84	0.000^†^
Fatigue	89	75	0.000^†^
Neck pain	106	74	0.000^†^
Headache or migraine	84	73	0.000^†^
Hip pain	77	71	0.000^†^
Chest pain	24	71	0.000^†^
Back pain	120	68	0.000^†^
Shoulder pain	81	69	0.000^†^
Wrist and hand pain	46	65	0.000^†^
Heartburn or indigestion	55	65	0.000^†^
Leg pain	15	47	0.002

* With “much more than usual” frequency.

† Approximate.

## Discussion

In the light of the answers to the present study, it was possible to confirm that most workers started working from home after the social isolation guidance that was issued as a consequence of the COVID-19 pandemic. In addition to this, a significant number of participants (84.2%) started working from home without any preparation, backup, evaluation, or support regarding health and safety at work provided by their employer. Often, in offices and usual work environments, the Occupational Health and Safety Department is responsible for choosing furniture and conducting a periodic analysis such as an Environmental Risks Prevention Program (ERPR) to assure adequate working conditions, including those related to lighting and noise exposure. Additionally, many workers had to live with other people in their homes, had to perform other tasks such as taking care of younger children and elderly people, and/or had to share their working space with other adults.

Some conditions, such as musculoskeletal and mental/emotional complaints, are the most frequently observed causes of work incapacity in Brazil.^17,18^ The results of this research show that participants emphasized aggravation of such conditions, in addition to other physical symptoms, such as sleeping problems, feelings of fatigue, headaches, migraines and ocular fatigue.

In a study in Italy,^5^ musculoskeletal disorders including low back pain (41.2%) were commonly reported. Half of the participants claimed to have worse cervical pain while working from home, besides low work satisfaction. In comparison, higher rates of low back pain were observed in Turkey,^4^ but there was no increase in other musculoskeletal conditions.

The public health crisis caused by the SARS CoV 2 pandemic has had significant social and economic repercussions. According to Del Boca et al.,^19^ in Italy the social distancing measures that obliged people to work and study from home led women, especially those with children between 0 and 5 years old, to spend more time on domestic chores and babysitting their kids. As for men, their overload was dependent on whether or not their partner was working from home.

A study conducted in Austria^6^ identified moderate to severe psychological impact in 43.3% of the participants, including depression, anxiety, and stress. This was particularly noted among women, older people, people with low levels of education, people highly concerned about their family members, those with low health self-assessment, and people who use the internet as their main source of information.

A longitudinal study comparing data from January 2018 and February 2020, during the shelter-at-home restrictions, observed more remote work at less formal workstations, reduced in-person social interactions during work and leisure time, and health behavior changes that were both negative (e.g., increased sitting and screen time) and positive (e.g., paying more attention to personal health). Negative impacts included more sedentary behavior on non-workdays, reduced sleep quality, increased mood disturbance, reduced quality of life, and reduced occupational health. While no worse dietary habits were found, red meat consumption was reduced; this could reflect reduced local availability of meat during the SARS CoV 2 pandemic period surveyed. Importantly, some factors did not worsen from before to during the COVID-19 shelter-at-home period, including lifestyle behaviors (e.g., workday sedentary behavior, physical activity, and most dietary habits) and some subscales of mood (e.g., fatigue), quality of life (e.g., general health), and occupational health (e.g., stress).^8^

Moretti et al. (2020)^5^ suggest that individuals should employ some measures related to organization of work at home to improve their performance: write a list of tasks for the day, make use of a space specifically reserved for work, and reduce sources of distraction. The employer could contribute to organizing this space by providing appropriate furniture (such as chairs, desks, and monitors) and offering professional postural guidance on site. During the period of social isolation, classes and videos were released via social media, some even featuring celebrities, without sufficient technical grounding, posing a potential threat to people’s health. These authors also called attention to the importance of organizations providing technical instruction on people’s working practices and adequate furniture, therefore stimulating people to be physically active and to achieve healthier working conditions regarding posture.

Moreover, the perceived health and well-being assessment using the WHO-5 instrument suggested an association between low scores and some conditions such as musculoskeletal disorders, feelings of fatigue, heartburn, indigestion, and leg pain. Therefore, it is important that care for these workers’ health is not limited to drug treatment for these symptoms, but also attends to causal factors, including those related to work organization, aiming at fully restoring workers’ health and well-being.

## Conclusions

Besides the positive aspects of home office, such as saving time spent on daily commuting, more contact with family, and productivity gains, the findings of this research confirm the importance of designing strategies and programs to preserve workers’ health and well-being. The balance between personal and professional life was already a well-studied subject before the SARS CoV 2 pandemic and now requires more effective approaches to support since the fine line between the different aspects of life may become even more subtle.

It is fundamental that companies develop strategies to follow-up on these workers, through their people management and/or occupational health departments, for example. The most frequent causes of illness and sick leave, such as musculoskeletal and mental/emotional conditions may be aggravated and the return to the regular work environment may occur under worse physical and emotional conditions.^7^ It is important to provide workers with support, by establishing frequent contact, revisiting goals and ways of monitoring them, and by involving workers in decision-making and priority-setting processes.

In a recent document (2020), The International Labour Organization^20^ (ILO) suggests that a certain level of flexibility should be established in terms of balancing personal and professional life and promoting health, as a means of preventing the worker from being available 24 hours a day, therefore assuring time for rest and personal life. The ILO also recommends focusing mainly on quality of work instead of quantity, clearly communicating expectations, and avoiding praising quick responses over appropriate ones. Some proposals tend to be less realistic, such as suggesting that workers should get a workspace free from interruptions and set boundaries between their professional activities and having contact with people who live with them. The ILO also advises workers of the importance of adopting a routine with healthy sleeping patterns, physical activity, and regular eating habits (without skipping meals and making good choices). Finally, it encourages leadership to identify changes in behavioral patterns in workers, such as occasional abusive consumption of alcohol and drugs, and to refer those who are exhibiting these habits to specialized support as soon as possible.

Working from home is an option for maintaining labor activities, even in social isolation, reducing the likelihood of unemployment. However, it requires both health and safety professionals to design strategies to protect workers’ health and well-being, avoiding aggravation of pre-established conditions and onset of others.
